# Multi-omics insights into the energy compensation of rumen microbiota of grazing yaks in cold season

**DOI:** 10.3389/fmicb.2024.1467841

**Published:** 2024-10-09

**Authors:** Jie Bai, Lijuan Tang, Yanliang Bi, Mingliang Li

**Affiliations:** ^1^Key Laboratory for Grassland Ecosystem of Ministry of Education, College of Pratacultural Science, Gansu Agricultural University, Lanzhou, China; ^2^National Engineering Research Center of Biological Feed, Feed Research Institute, Chinese Academy of Agricultural Sciences, Beijing, China; ^3^Livestock and Poultry Genetic Resources Protection and Utilization Center in Qinghai Province, Xining, China

**Keywords:** Yak, rumen microbiota, VFAs transport, extremely environmental, adaptation

## Abstract

**Background:**

The ability of yaks to adapt to the extreme environment of low temperatures and hypoxia at cold seasons on the Qinghai-Tibet Plateau (QTP) is related to the host genome; however, the convergent evolution of rumen microbiomes in host adaption is unknown.

**Methods:**

Here, we conducted a multi-omics study on the rumen fluid of grazing yaks from warm (July) and cold (December) seasons on the QTP to evaluate the convergent evolution of rumen microbiomes in the adaptation of grazing yaks to cold-seasons environments.

**Results:**

The results showed that grazing yaks at cold seasons had higher fibrolytic enzyme activities and volatile fatty acids (VFAs) concentrations, and the relative abundance of Firmicutes and the ratio Firmicutes to Bacteroidetes was significantly higher than that of yaks at warm seasons. Macrogenomic analyses showed that genes involved in forming VFAs and arginine were significantly enriched in cold-season yaks. Transcriptome analyses of the rumen epithelium showed that 72 genes associated with VFAs absorption and transport were significantly upregulated in cold-season yaks. Metabolomic analyses showed that the levels of ornithine, related to efficient nitrogen utilization, were significantly upregulated in cold-season yaks.

**Conclusion:**

The synergistic role of rumen microbiomes in the adaptation of grazing yaks to extreme environments at cold seasons was revealed by multi-omics study.

## Introduction

The demanding conditions present in cold-season environments - low temperatures, lack of oxygen, and limited food resources - present a significant challenge to the survival of animal populations (Wang et al., 2024). The gastrointestinal microbiome offers crucial functions to the host, including energy balance, regulation of immunity, food fermentation, physical development, and prevention of diseases (Mao et al., 2015; Bai et al., 2022; Han et al., 2022). The gastrointestinal microbiome is closely associated with host adaptation (Huws et al., 2018; Guo et al., 2021; Mizrahi et al., 2021). Understanding the potential role of gastrointestinal microbiota in animal adaptation to challenging cold-season environments is crucial to investigate the convergent evolution of gastrointestinal microbiota.

The variety and diversity of microbial colonization in the gastrointestinal tract of animals are determined by natural selection between the host and its living environment. Previous studies on piglets, partridges, pikas, Tibetan chickens, Tibetan pigs, and rhesus macaques have confirmed that gastrointestinal microbiomes positively affect adaptation to harsh environments (Zhou et al., 2016; Li et al., 2018; Liu et al., 2021; Zhang et al., 2022; Tang et al., 2023; Zhao F. F. et al., 2023; Zhao J. S. et al., 2023). Volatile fatty acids (VFAs) supplied by the gastrointestinal microbiome play a vital role in host energy harvesting (Ganal-Vonarburg et al., 2020; Kimura et al., 2020; Nilsen et al., 2020; Zietek et al., 2021). Previous research suggests that alterations in the gastrointestinal microbiome of animals can lead to changes in their function (Huws et al., 2018; Sha et al., 2024); however, the function of gastrointestinal microbiomes in response to extreme environments is unknown.

The yak is an endemic breed that lives at high altitudes. Approximately 90% of the world’s yaks live in the Qinghai-Tibet Plateau (QTP) region of China, where they provide milk, meat, wool, and fuel to the Tibetan people (Wang et al., 2024). Therefore, local herders call them all-round livestock and plateau boats. Previous research has demonstrated that the ruminal microbial community composition in yaks is affected by diet, age, season, activity area, and health status (Ahmad et al., 2020; Guo et al., 2021; Han et al., 2022). However, no studies have yet reported on the interactions between functional genes and metabolite composition of the rumen microbiota of yaks during cold-season adaptation. In addition, adaptation mechanisms for the genome and physiology of grazing yaks at cold season have been widely reported (Ishizaki et al., 2005; Shao et al., 2010; Qiu et al., 2012); however, the convergent evolution of rumen microbiota in host adaptation is currently unknown. Therefore, we conducted investigations on the microbiome, macrogenome, and metabolome of rumen fluid and the transcriptome of rumen epithelium from warm- and cold- season grazing yaks on the QTP to evaluate the synergistic role of rumen microbiomes in yak adaptation to harsh environments at cold season. These findings offer fresh perspectives on the convergent evolution of gut microbes in animals adapted to extreme cold-season environments.

## Materials and methods

### Experimental design and sampling

All trial procedures of this study have been approved by the Animal Ethics Committee of Gansu Agricultural University (GAU-LC-2020-27). The 12 healthy male yaks, aged five years and with an average body weight of 236.17 ± 7.36 kg, were sourced from a grazing area situated at an altitude of 3,100 m on the QTP. The yaks were managed using traditional natural grazing techniques. They were allocated randomly into two cohorts to reflect seasonal variations-warm (July) and cold (December), with each group consisting of six yaks. These animals grazed together in the same field. Sample collections occurred in July and December. Yaks were subsequently slaughtered and rumen fluid and rumen epithelial tissues were collected. The rumen content (70 mL) was collected from each yak and filtered using four layers of gauze. The collected rumen fluid was divided into three parts: one part was placed into a 10 mL lyophilized tube for DNA extraction, another part was loaded into a 10 mL lyophilized tube for metabolite identification, and the third part was placed into a 50 mL centrifuge tube to determine rumen fermentation parameters. Following sample dispensing, the samples were transferred using liquid nitrogen tanks to the laboratory where they were then stored in an ultra-low temperature refrigerator (−80°C) for subsequent analysis. The tissue of the rumen ventral capsule was cut and promptly rinsed with PBS buffer to separate the epithelial tissue, which was then immediately stored in liquid nitrogen for the extraction of total RNA from the ruminal epithelium. Furthermore, six test plots measuring 0.5 × 0.5 meters were randomly established within each experimental area to document the plant species. Subsequently, the vegetation in the plots was trimmed to 5 centimeters from the ground level, gathered in cloth bags, and taken back to the laboratory to assess the nutritional value of the forage.

### Determination of rumen histomorphology in grazing yaks

Five pieces of tissues measuring 2.5 × 2.5 cm were clipped from the abdominal sac area of each yak separately. Rumen ventral sac tissues were soaked in paraformaldehyde (4%) for 24 h, subsequently dehydrated, made transparent, waxed, embedded, sectioned, and stained. Hematoxylin (Wuhan Sevier Biotechnology Co., Ltd.) and eosin (Hefei Bomei Biotechnology Co., Ltd.) were used for the staining. The sections were observed using a Pannoramic 250 (3DHISTECH) scanner. The following were measured using the CaseViewer section analysis system: rumen nipple height, nipple width, stratum corneum, granular layer, spinous layer, base layer, and muscle thickness.

### Determination of the nutritional quality of herbage

Forage samples from two seasons were dried at 65°C for 48 h and reduced to approximately 1 mm size. The dry matter (DM), crude protein (CP), ether extract (EE), neutral detergent fiber (NDF), and acid detergent fiber (ADF) contents were determined under laboratory conditions. The DM, CP, and EE contents were analysed according to the method outlined by AOAC (2002), whilst the NDF and ADF contents were evaluated through employment of a fully automated ANKOM A2000i instrument.

### Determination of VFAs and fibrolytic enzyme activity

VFAs were analysed by means of gas chromatography (chromatograph SP-3420A, Beifenrili Analyzer Associates, Beijing, China), following the procedure outlined in Erwin et al. (1961). Carboxymethyl cellulase, avicelase, xylanase, and acetylesterase levels were measured using xylanase, CMCase, avicelase, and AE enzyme immunoassay kits (Jiangsu Jingmei Bio-Technology Co., Ltd., 96T, 3 U/L–80 U/L), respectively.

### High-throughput sequencing and analysis

DNA was extracted from yak rumen fluid using the E.Z.N.A.^®^ Stool kit, in accordance with manufacturer instructions provided by Omega BioTEK in Norcross, GA. The quality and purity of the extracted DNA were evaluated via 1% agarose gel electrophoresis and spectrophotometry, using Thermo Scientific’s NanoDrop 2000C equipment. The bacterial 16S rRNA gene’s V3-V4 region was amplified using the 338F primer (5′-ACTCCTACGGGGAGGCAGCAG-3′) and the 806R primer (5′-GGACTACHVGGGTWTCTAAT-3′). The PCR products were subsequently observed by a 1% agarose gel electrophoresis and purified utilizing an Agencourt^®^ AMPure^®^ XP (Beckman Coulter) Nucleic Acid Kit. The sequencing libraries were constructed with the PCR products, followed by high-throughput sequencing using Illumina MiSeq PE300.

The raw sequences obtained were spliced and filtered using QIIME1 (v.1.8.0), Pear (v.0.9.6), and Vsearch (v.2.7.1) softwares to obtain high-quality data. Subsequently, the high-quality data were compared with the Gold Database to obtain data that could be used for subsequent analyses. Finally, we utilized Vsearch (v.2.7.1) software to analyze the sequencing data and grouped the sequences into a single class of OTUs based on a similarity threshold of over 97%. Using the RDP Classifier algorithm, each OTU was compared with the Silva128 database to obtain classification information. Finally, OTU data were standardized using the minimum draw flat method for further microbial α-diversity indices.

### Metagenomic sequencing and analysis

The DNA samples under test were randomly fragmented into small pieces of roughly 300 bp using a Covaris M220. TruSeq DNA Sample Preparation Kit (Illumina, San Diego, CA) was employed to establish individual sequencing libraries, and bipartite sequencing was performed using the Illumina NovaSeq PE150 Sequencing Platform. The fastp v0.20.0 software was utilized to trim and obtain high-quality sequences. All reads were aligned to yak DNA sequences using BWA v0.7.9 to detect and exclude any reads with significant similarity (Li et al., 2014). The optimized sequences were spliced and assembled using MEGAHIT v1.1.2, and contigs with lengths greater than 800 bp were filtered out for further analysis.^1^

ORF prediction of the filtered contigs was performed using Prodigal (https://github.com/hyattpd/prodigal/wiki). The gene sequences predicted from all samples were clustered using CD-HIT v4.6.1,^2^ based on an identity greater than 95% and coverage greater than 90%. The non-redundant gene set was constructed using the longest sequences from each cluster as their representative sequences. To determine gene abundance, quality-filtered sequence reads were compared with those with > 95% identity using Bowtie2 across each sample (Fu et al., 2012). The non-redundant gene set’s representative sequences underwent alignment with NR v2021.11, eggNOG v4.5.1, and KEGG v94.2 databases using Diamond v0.8.35. Subsequently, species abundance, gene-related functions and KEGG functions were attained (Xie et al., 2011). The abundance of carbohydrate-active enzymes (CAZymes) was determined by aligning representative sequences from the non-redundant gene set with the CAZy v5.0 database^3^ using hmmscan.

### Transcriptome sequencing and analysis

RNA was extracted from grazing yaks’ rumen epithelial tissue through the TRIzol technique (Invitrogen, CA). RNA quality and purity were assessed using a spectrophotometer (NanoDrop Thermo Scientific, DE), while extracted RNA integrity was evaluated through an Agilent 2100 spectrophotometer (Agilent Technologies, CA). The examined RNA samples were employed to fabricate distinct cDNA collections with the VAHTS Universal V6 RNASEQ Library Prep Kit (Illumina) following the manufacturer’s guidelines. The commodities were then refined with a VAHTSTM DNA Clean Beads Kit (N411-03). The formed collections were analysed using Illumina NovaSeq 6000 (PE150).

Firstly, raw sequences were filtered through in-house Perl scripts. After this, the raw sequences were quality-clipped with Trimmomatic v0.33, resulting in obtaining high-quality sequences. Subsequently, the alignment of the yak genome was carried out using STAR v2.5.2b. Cufflinks v2.1.1 was utilized to assemble the comparison results. Measurement of transcription and gene expression levels was performed using FPKM. Differential gene expression was analysed using DESeq v1.10.1 data analysis methods, adhering to fold change ≥ 2 and FDR < 0.01. Subsequently, GOseq v1.22 was employed to conduct functional enrichment analyses of GO and KEGG, specifically concerning differentially expressed genes.

### Metabolomic analysis of rumen fluid

The samples of rumen fluid were removed from the ultra-low temperature fridge (−80°C) and left to thaw at room temperature (4°C). 500 μL of each sample was taken and a CAN:MeOH (v:v = 1:1) solution was added. After 30 seconds of shaking (using a vortex shaker), they were sonicated for 10 min (sonicator, PS-60AL) and subsequently left to settle at −20°C for 1 h. The supernatant was then centrifuged at 13,000 rpm at 4°C for 15 min. The supernatant underwent a 15-min ultra-high-performance liquid chromatography analysis (LC-30, Shimadzu). The ACQUITY UPLC HSS T3 column with a dimension of 2.1 × 100 mm and a particle size of 1.8 μm was used with water as the eluent. An injection volume of 2 μL was applied, and the flow rate was set at 0.3 mL/min while the column temperature was held at 50°C. Rumen fluid samples were collected using high-resolution mass spectrometry (Triple TOF 5600) in both positive and negative ion modes. The raw data were processed for peak detection, extraction, alignment, and integration using the Allwegene Company program (Beijing, China) after being converted to the mzML format using ProteoWizard. Metabolites were identified using the Allwegene database with a threshold of 0.7. For further data analysis, the raw data underwent additional steps. Firstly, peaks with missing values exceeding 50% of the sample were eliminated. Secondly, the missing values in the raw data were filled by half of the minimum value. Finally, the data passed through quality control by excluding mass spectra with metabolic profiles exhibiting a relative standard deviation of over 30%.^4^ Mapping of all differential metabolites to the biochemical pathways in which they participated was performed by searching the KEGG database, metabolite enrichment, and pathway analysis.

### Statistical analysis

The data analysis was conducted using SAS 9.2 statistical software. Forage nutritional quality, rumen fermentation parameters, relative abundance of bacteria at the phylum and genus levels, fibrolytic enzyme activities, abundance of CAZyme genes, and rumen tissue structure parameters were analyzed using the T-test. A significant difference was noted when *P* < 0.05, while *P* > 0.05 was considered insignificant. The significant upregulation of ruminal epithelial transcription genes was standardized at *P*-adj < 0.05. The screening for significantly different metabolites was at VIP ≥ 1, *P* < 0.05, and FC < 0.67 or > 1.5. Differentially expressed genes in different metabolic pathways were compared using the Wilcoxon test. Graphics were created using R software (version 4.0.2).^5^

## Results

### Differences in the nutrient composition of herbage at warm and cold seasons

The warm season herbage had a significantly higher CP and EE contents compared to the cold season herbage (Table 1; *P* < 0.05). In contrast, the NDF and ADF contents of cold season herbage were significantly higher than those of warm season herbage (*P* < 0.05). No notable difference in the DM and OM contents of herbage was observed between warm and cold seasons (*P* > 0.05).

**TABLE 1:** Differences in herbage nutrient composition between warm and cold seasons.

Item	Season		SEM	*P*-value
	W	C		
DM	92.8	93.26	0.2229	0.3178
CP	10.86	5.39	0.8527	<0.0001
EE	1.56	0.88	0.1055	<0.0001
NDF	42.91	58.73	2.4722	<0.0001
ADF	28.09	33.65	0.9997	0.0006
OM	89.72	89.62	0.1367	0.7416

Season: W, warm season; C, cold-season. CP, crude protein; EE, ether extract; NDF, neutral detergent fiber; ADF, acid detergent fiber; DM, dry matter; OM, organic matter.

### Differences in VFAs and fibrolytic enzyme activities in yak rumen at warm and cold seasons

The concentrations of TVFA and the proportions of acetate and propionate in the rumens of yaks at cold season were significantly higher than those at warm season (Table 2, *P* < 0.05). However, the proportions of butyrate and isovalerate in the rumens of yaks at warm season were significantly higher than those at cold season (*P* < 0.05). Furthermore, the activities of avicelase, xylanase, and carboxymethyl cellulase in the rumen of yaks at cold seasons were significantly higher than those at warm seasons (Table 2, *P* < 0.05). The activities of acetylesterase in the rumen of yaks at warm and cold seasons did not display significant differences (*P* > 0.05).

**TABLE 2:** Differences in rumen fermentation parameters and fibrolytic enzyme activities between warm and cold seasons.

Item	Season		SEM	*P*-value
	W	C		
**Rumen fermentation parameters**				
Total VFA (mmol/L)	42.08	73.58	4.813	<0.0001
Acetate (%)	59.96	63.01	0.7645	0.0384
Propionate (%)	19.18	23.15	0.8636	0.0124
Butyrate (%)	18.21	11.91	1.0437	<0.0001
Isobutyrate (%)	0.82	0.77	0.0434	0.5899
Valerate (%)	0.78	0.65	0.0405	0.1164
Isovalerate (%)	1.03	0.49	0.085	<0.0001
**Fibrolytic enzyme activities**				
Avicelase (IU/L)	8.53	24.42	2.9328	0.0012
Xylanase (U/L)	32.11	38.94	1.7244	0.0405
Carboxymethyl cellulase (IU/L)	170.54	244.02	11.8086	<0.0001
Acetylesterase (U/L)	43.85	39.67	2.5955	0.4474

VFA, volatile fatty acids. Season: W, warm season; C, cold-season.

### Differences in the rumen tissue structure of yaks at warm and cold seasons

The nipple height, nipple width, stratum corneum thickness, granular layer thickness, Spinous layer thickness, base layer thickness, and muscle thickness of the yak rumen were significantly higher at cold season than at warm season (Supplementary Table 1 and Supplementary Figure 1, *P* < 0.05).

### Differences in the microbial community composition of yak rumen at warm and cold seasons

**FIGURE 1:**
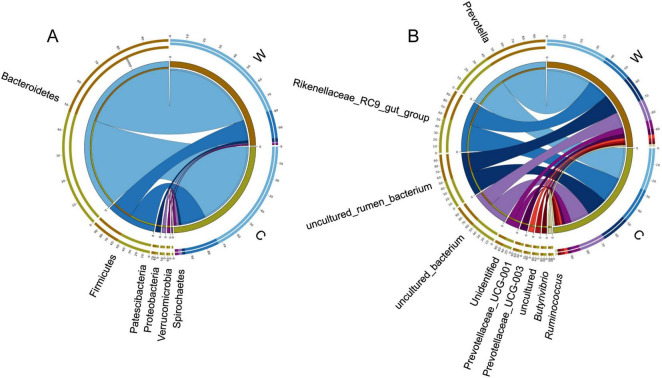
Differences in rumen bacterial community composition between yaks at warm and cold seasons. Relative abundance of rumen bacteria at the phylum **(A)** and genus **(B)** level.

At the phylum level, rumen bacteria mainly included Bacteroidetes, Firmicutes, Proteobacteria, Patescibacteria, and Verrucomicrobia (Figure 1A). The relative abundance of Firmicutes and the ratio Firmicutes to Bacteroidetes were significantly higher at cold season than at warm season (Supplementary Table 2, *P* < 0.05). The relative abundance of Bacteroidetes was significantly higher at warm season than at cold season (Supplementary Table 2, *P* < 0.05). At the genus level, rumen bacteria mainly included *Prevotella*, *Rikenellaceae* RC9 gut group, *Prevotellaceae* UCG-001, and *Prevotellaceae* UCG-003 (Figure 1B). The relative abundance of *Prevotella* and *Prevotellaceae* UCG-001 were significantly higher at warm season than at cold season (Supplementary Table 2, *P* < 0.05). The relative abundance of *Lachnospiraceae* XPB1014 group, *Lachnospiraceae* AC2044 group, and *Clostridiales* bacterium Firm 14 were significantly higher at cold season than at warm season (*P* < 0.05).

### Differences in CAZymes and KEGG pathway of yak rumen at warm and cold seasons

The relative abundance of carbohydrate-binding modules (CBMs), glycoside hydrolases (GHs), glycosyltransferases (GTs), polysaccharide lyases (PLs), and CAZymes in yak rumens at cold season were significantly higher than that at warm season (Supplementary Table 3, *P* < 0.05). The GH, GT, PL, and CBM families associated with polysaccharide degradation were compared (Supplementary Figure 2). The relative abundance of *GH5*, *GH43*, *GH66*, *GH101*, *GH6*, *GT9*, *GT30*, *PL*21, and *CBM5* in the yak rumen at cold season were significantly higher than that at warm season (*P* < 0.05). The relative abundance of *GH26* in the yak rumen at warm season was significantly higher than that at cold season (*P* < 0.05).

**FIGURE 2:**
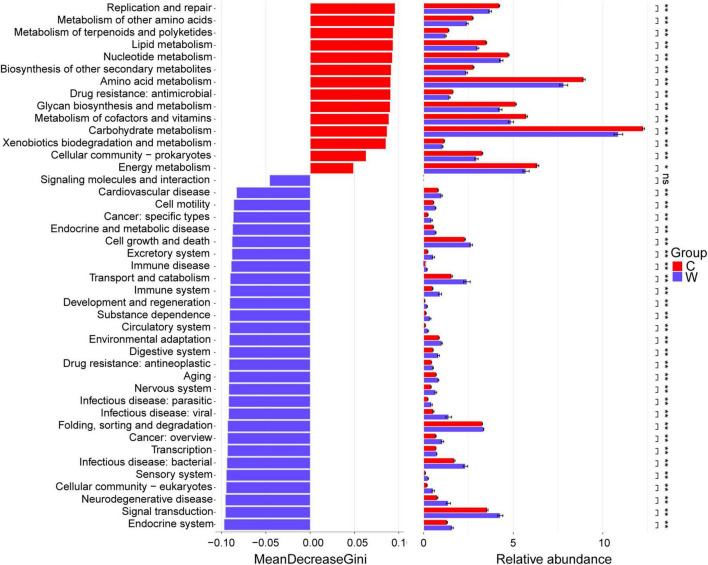
Differential KEGG pathways between yaks at warm and cold seasons at level 2. Season: W, warm season; C, cold-season.

At level 2 (Figure 2), Replication and repair, metabolism of other amino acids, metabolism of terpenoids and polyketides, lipid metabolism, nucleotide metabolism, biosynthesis of other secondary metabolites, amino acid metabolism, drug resistance: antimicrobial, glycan biosynthesis and metabolism, metabolism of cofactors and vitamins, carbohydrate metabolism, xenobiotics biodegradation and metabolism, cellular community-prokaryotes, energy metabolism, and translation were enriched in the cold season yaks (*P* < 0.05). Endocrine system, signal transduction, neurodegenerative disease, cellular community-eukaryotes, sensory system, infectious disease: bacterial, transcription, cancer: overview, folding, sorting and degradation, infectious disease: viral, infectious disease: parasitic, nervous system, aging, drug resistance: antineoplastic, digestive system, environmental adaptation, circulatory system, substance dependence, development and regeneration, immune system, transport and catabolism, immune disease, excretory system, cell growth and death, endocrine and metabolic disease, cancer: specific types, cell motility, and cardiovascular disease in warm season yaks (*P* < 0.05).

At level 3 (Supplementary Figure 3), 7 and 22 metabolic pathways were enriched in the warm and cold season yaks (*P* < 0.05), respectively. Endocytosis, pathways of neurodegeneration_multiple diseases, autophagy_animal, amyotrophic laterals clerosis, and coronavirus disease_COVID_19 were the top five pathways for warm season yaks; Ribosome, aminoacyl_tRNAbiosynthesis, amino sugar and nucleotide sugar metabolism, purineme tabolism, and homologous recombination were the top five pathways for cold season yaks.

**FIGURE 3:**
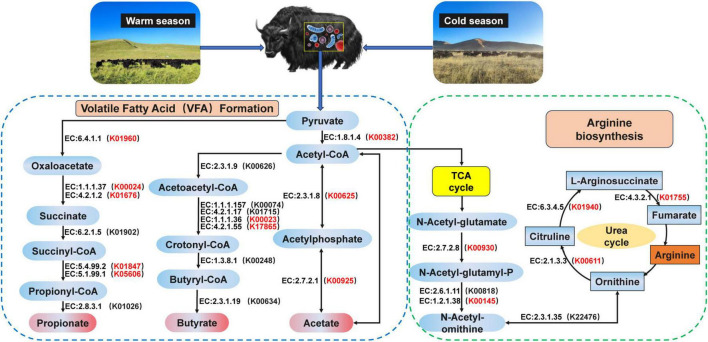
Reconstruction of the metabolic pathway associated with VFAs formation and arginine biosynthesis between warm and cold seasons. The red KO in parentheses indicates enrichment in cold season yaks. The metabolic pathway for VFAs formation is referenced from Zhang et al. (2016). The metabolic pathway for arginine biosynthesis is referenced from Guo et al. (2021).

In addition, we analyzed the metabolic pathway from pyruvate to acetate, propionate, and butyrate and found that the pathway mainly involved 10 encoded enzymes (Figure 3). K00382, K00625, and K00925 of the yak rumen were found to be significantly enriched at cold season in the pyruvate-to-acetate metabolic pathway (*P* < 0.05). K00023 and K17865 of the yak rumen were significantly enriched at cold season in the pyruvate to butyrate metabolic pathway (*P* < 0.05). K01960, K00024, K01676, K01847, and K05606 of the yak rumen were significantly enriched at cold season in the pyruvate to propionate metabolic pathway (*P* < 0.05). The metabolic pathways of ornithine were further analyzed (Figure 3), and we found that the pathways mainly involved 7 encoded enzymes. In these pathways, K00930, K00145, K01940, K00611, and K01755 of the yak rumen were significantly enriched at cold season (*P* < 0.05).

### Differences in VFAs transport and absorption in the ruminal epithelium of yaks at warm and cold seasons

**FIGURE 4:**
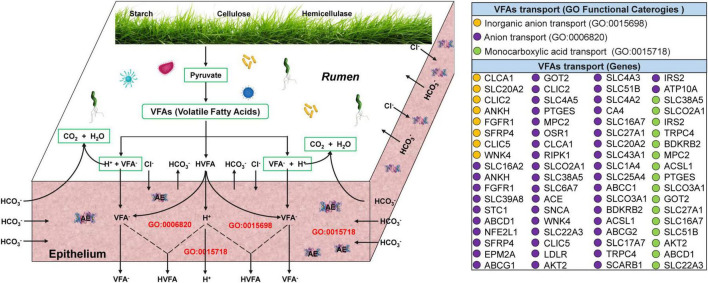
Transcriptome analyses of genes related to VFAs transport and absorption across the ruminal epithelium in yaks at warm and cold seasons. The three functional categories (anion, inorganic anion, and monocarboxylic acid transport) associated with VFAs transport and absorption were referred from Georgi et al. (2014) and Aschenbach et al. (2011). The 72 genes associated with anion (purple circles), inorganic anion (brown circles), and monocarboxylic acid transport (green circles) were significantly upregulated (*q*-value < 0.05; Supplementary Table 4).

Transcriptome analysis of the yak rumen epithelium revealed that 72 genes associated with the transport and absorption of VFAs were significantly upregulated at cold season (Figure 4 and Supplementary Table 4). Of these, 8 (*CLCA1*, *SLC20A2*, *CLIC2*, *ANKH*, *FGFR1*, *SFRP4*, *CLIC5*, and *WNK4*), 48 (*PTGES*, *MPC2*, *OSR1*, *CLCA1*, *RIPK1*, *SLCO2A1*, *SLC38A5*, *SLC6A7*, *ACE*, *SNCA*, *WNK4*, *SLC22A3*, *CLIC5*, *LDLR*, *AKT2*, *SLC4A3*, *SLC51B*, *SLC4A2*, *CA4*, *SLC16A7*, *SLC27A1*, *SLC20A2*, *SLC43A1*, *SLC1A4*, *SLC25A4*, *ABCC1*, *SLCO3A1*, *BDKRB2*, *ACSL1*, *ABCG2*, *SLC17A7*, *TRPC4*, *SCARB1*, *IRS2*, *ATP10A*, *SLC16A2*, *ANKH*, *FGFR1*, *SLC39A8*, *STC1*, *ABCD1*, *NFE2L1*, *SFRP4*, *EPM2A*, *ABCG1*, *GOT2*, *CLIC2*, and *SLC4A5*), and 16 genes (*SLC38A5*, *SLCO2A1*, *IRS2*, *TRPC4*, *BDKRB2*, *MPC2*, *ACSL1*, *PTGES*, *SLCO3A1*, *GOT2*, *SLC27A1*, *SLC16A7*, *SLC51B*, *AKT2*, *ABCD1*, and *SLC22A3*) were significantly enriched in inorganic anion, anion, and monocarboxylic acid transport, respectively.

### Differences in yak rumen metabolites at warm and cold seasons

**FIGURE 5:**
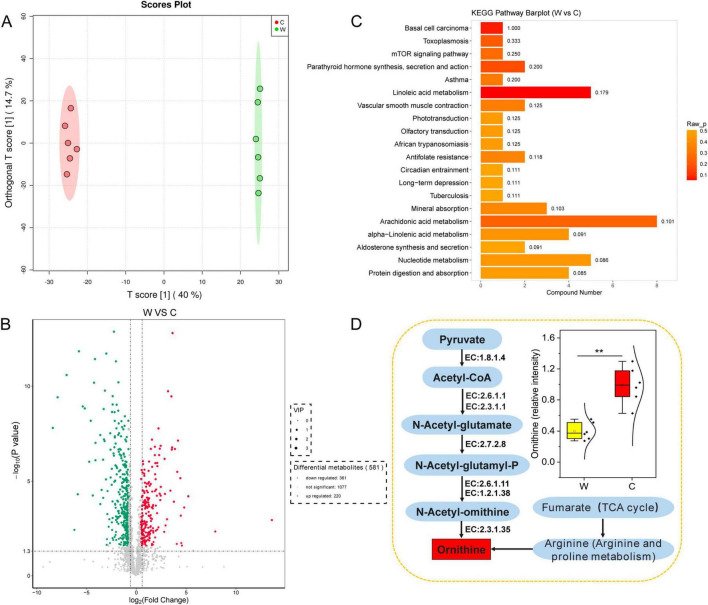
Abundance differences in rumen fluid metabolites annotated by the KEGG database between yaks at warm and cold seasons. **(A)** OPLS-DA analysis showed that rumen metabolite profiles were distinct between yaks at warm and cold seasons. The ellipse borders represent 95% confidence interval. **(B)** Volcano map of differential rumen metabolite profiles between yaks at warm and cold seasons; red and green indicate that the rumen metabolite profiles were significantly upregulated at warm and cold seasons, respectively. **(C)** KEGG enrichment histogram of yaks at warm and cold seasons. The numbers in the columns represent rich factors, and the rich factor represents the ratio of the number of differential metabolites in the pathway to the number of metabolites annotated in the pathway. **(D)** Difference in the metabolic pathway of ornithine between yaks at warm and cold seasons.

The OPLS-DA model (Figure 5A) revealed a significant difference (Supplementary Figure 4) in the rumen metabolites at warm and cold seasons (R^2^X = 0.99, Q^2^ = 0.97). We found 581 significantly different metabolites (VIP ≥ 1, *P* value < 0.05 and fold change < 0.67 or > 1.5) between warm and cold season yaks, of which 220 metabolites were significantly upregulated and 361 metabolites were significantly downregulated (Figure 5B). KEGG enrichment analysis was used to determine the metabolic pathways of the significantly different metabolites (Figure 5C and Supplementary Figure 5). The pathways for tuberculosis, toxoplasmosis, protein digestion and absorption, mTOR signaling pathway, mineral absorption, linoleic acid metabolism, and alpha-inoleic acid metabolism were significantly upregulated in the warm season yaks, and nucleotide metabolism and arachidonic acid metabolism were significantly upregulated in the cold season yaks. In addition, ornithine was significantly upregulated in the cold season yaks (Figure 5D).

## Discussion

During the cold season on the QTP, the CP content in forage decreases, while the NDF and ADF content increase, further limiting the nutritional supply of forage for yaks. Many species of animals living at cold season have adapted to this extreme environment by consuming more energy through accelerated metabolism to maintain normal survival, compared to animals living at warm season (Li et al., 2017; Liu et al., 2022). The rumen of ruminants contains numerous microorganisms (Zhao et al., 2017; Stewart et al., 2019). Ruminants rely on rumen microbes to degrade cellulose, hemicellulose, and lignin in forage to produce VFAs that provide energy (Gharechahi et al., 2021; Xue et al., 2022). Therefore, we hypothesized that these adaptive evolutions occurred in the yak rumen, with several microorganisms playing a major role. To test this hypothesis, we analyzed cellulase activity, VFAs concentration, and rumen microbiota of yaks at both warm and cold seasons and found that the cellulase activity (avicelase, xylanase, and carboxymethyl cellulase) and VFAs concentration in the rumen of yaks grazing at cold season were significantly higher. In addition, the relative abundance of Firmicutes, *Lachnospiraceae* XPB1014 group, *Lachnospiraceae* AC2044 group, and *Clostridiales* bacterium Firm 14 significantly increased at cold season, whereas the relative abundance of Bacteroidetes significantly decreased. The Firmicutes are responsible for a number of essential functions within the digestive system, including energy conversion and harvesting. In contrast, the Bacteroidetes are involved in a range of processes, including carbohydrate degradation and protein hydrolysis (Turnbaugh et al., 2006; Chevalier et al., 2015). The *Lachnospiraceae* XPB1014 group, *Lachnospiraceae* AC2044 group, and *Clostridiales* bacterium Firm 14 were observed to produce VFAs (Thomas et al., 2011; Xue et al., 2020; Sha et al., 2024). These results suggest that, under cold-season grazing conditions, yak rumen microorganisms can enhance the breakdown and utilization efficiency of cellulose, enabling yaks to more effectively obtain energy and adapt to extreme environmental conditions such as low temperatures and low oxygen levels. The metabolic pathways for the production of VFAs were further analyzed, and 8 enzymes in the metabolic pathways from pyruvate to propionate and acetate were found to be significantly enriched in the rumen of grazing yaks at cold season, which contributed to the production of propionate and acetate, which was consistent with the fact that the concentration of propionate and acetate was significantly higher at cold season. The findings indicated that rumen microorganisms in yak, which graze during the cold season, produce a greater quantity of VFAs to provide the host with additional energy, thereby enabling the host to adapt to the extreme environmental conditions that prevail during the cold season.

Most VFAs produced by fermentation in the rumen are directly absorbed and transported across the rumen epithelium, playing a pivotal role in the regulation of host energy balance (Aschenbach et al., 2011; Liu et al., 2020). In this study, VFAs concentration in the rumen of grazing yaks at cold season significantly increased. Therefore, we hypothesized that the rumen epithelium of grazing yaks at cold season absorbs and transports VFAs more efficiently to provide more energy to the host to adapt to the extreme environments at cold seasons. A large number of keratinized papillae are distributed on the rumen epithelium, and the presence of keratinized papillae enlarges the contact area between the rumen contents and rumen epithelium, which is beneficial for nutrient absorption and ion transport (Sun et al., 2021). In this study, the width and height of the rumen papillae of grazing yaks at cold seasons were significantly increased, suggesting that the rumen papillae of grazing yaks at cold seasons may more efficiently absorb and transport VFAs. Zhang et al. (2016) investigated the uptake and transport capacity of VFAs in yaks and cattle using rumen epithelial transcriptome sequencing and found that 36 genes related to the uptake and transport of VFAs in yaks were significantly upregulated. In the present study, transcriptome analysis of rumen epithelial tissue revealed a significant upregulation of 72 genes related to the absorption and transport of VFAs in the rumen epithelium of grazing yaks at cold seasons. Two forms of VFAs are present in the rumen: the ionized form (VFA^–^) and the protonated form (HVFA). The two types of VFAs have different absorption and transport processes in the rumen epithelium, with the protonated form being absorbed mainly by free diffusion and the ionized form being absorbed mainly by active transport (Wu et al., 2022; Xu et al., 2023). The absorption of VFA^–^ during active transport occurs via exchange with anions such as HCO_3_^–^ and Cl^–^ (Georgi et al., 2014). Gabel et al. (2001, 2002) showed that the absorption ability of VFA^–^ of the rumen epithelium is positively correlated with the expression of anion (HCO_3_^–^ and Cl^–^) carriers. CLCA1 is a key Cl^–^ channel in the rumen epithelium that plays a vital role in the exchange of VFA^–^ with HCO_3_^–^ and Cl^–^ (Guo et al., 2022). In this study, CLCA1 was significantly enriched in cold-season yaks, suggesting that the rumen epithelium of grazing yaks at cold season may absorb and transport VFAs and provide more energy to the host to adapt to the extreme environments at cold seasons.

We analyzed the functional enrichment of differential metabolites at warm and cold seasons and found that the enrichment scores of the arachidonic acid and nucleotide metabolism pathways were higher in cold-seasons yaks. Previous studies have demonstrated that arachidonic acid plays a critical role in enhancing animals’ cold resistance, immune function, skin and hair health, energy metabolism, fat mobilization, and antioxidant capacity (Yang et al., 2020; He et al., 2021; Kaur et al., 2022; Yan et al., 2022; Guo et al., 2023). In this study, we observed a significant increase in the arachidonic acid content in the rumen of yaks during the cold season. These findings suggest that arachidonic acid aids yaks in adapting to cold-season challenges by improving cold resistance, boosting immune function, promoting energy mobilization, and maintaining skin and hair health. During cold seasons, the enrichment of nucleotide metabolic pathways in the yak rumen may facilitate the provision of additional substrates necessary for DNA replication and repair (Zhao et al., 2022). In addition, this study found that ornithine levels were significantly upregulated in cold-season yaks. Functional enrichment analysis of yak rumen microorganisms revealed that genes involved in ornithine biosynthesis were significantly enriched in cold-season grazing yaks. During the cold season, yaks graze on natural grasslands with low nitrogen content of the pasture. Enrichment of genes in the ornithine synthesis pathway was found, resulting in accelerated recirculation of urea and reduced excretion to the outside of the body. The efficient nitrogen utilization mechanism of yaks adapted to the cold season nutritional stress on the QTP was further confirmed (Guo et al., 2021). Overall, the rumen microbial metabolite changes were consistent with the macrogenomic results, which confirmed the adaptation of yaks to cold-season environments.

## Conclusion

**FIGURE 6:**
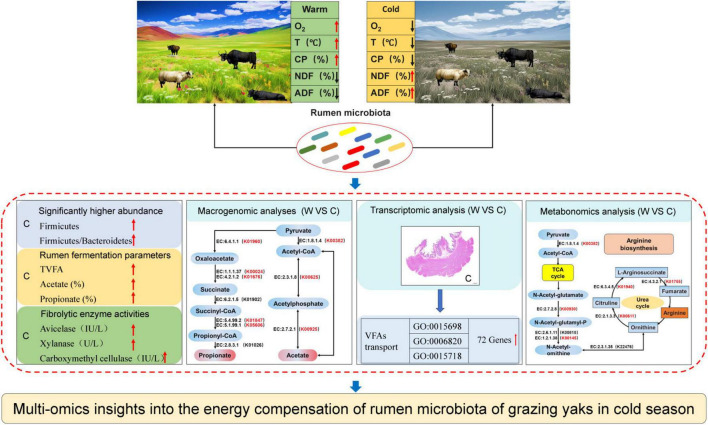
Study design and main results.

This study revealed the synergistic role of rumen microbes in the adaptation of yaks to harsh environments at cold seasons (Figure 6). Grazing yaks at cold seasons had higher fibrolytic enzyme activities, and the relative abundance of microbes related to fiber degradation and VFAs production was significantly higher than that of yaks at warm seasons. Genes associated with VFAs formation, absorption, and transport within the rumen epithelium were significantly enriched in cold-season yaks. The provision of these energy substrates enables the host to achieve enhanced energy compensation during the cold seasons. Furthermore, our findings indicate that the enrichment of genes in the arginine synthesis pathway during the cold season resulted in the acceleration of urea cycling and a reduction in excretion to the exterior of the body. This provides further confirmation of the mechanism of efficient nitrogen utilization in yaks adapted to the cold season nutrient stress on the QTP.

## Data Availability

The sequencing raw data were deposited in the NCBI BioProject database under the accession number PRJNA1167791.

## Footnotes

1
https://github.com/voutcn/megahit

2
http://www.bioinformatics.org/cd-hit/

3
http://www.cazy.org/

4
https://fiehnlab.ucdavis.edu/projects/fiehnlib

5
https://CRAN.R-project.org
