# Supplementary material for: Multi-omics insights into the energy compensation of rumen microbiota of grazing yaks in cold season
Source: Front Microbiol. 2024 Oct 9;15:1467841. doi: 10.3389/fmicb.2024.1467841 (PMC11496799; doi:10.3389/fmicb.2024.1467841)
Supplement: Supplementary file 2 [file Data_Sheet_1.docx]

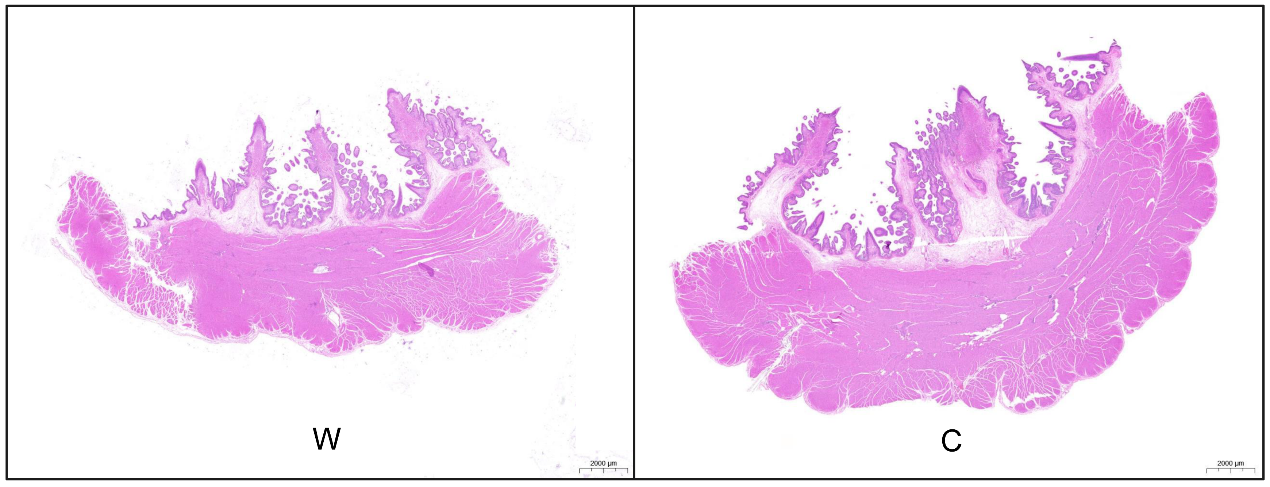


**Figure S1** Comparison of rumen histomorphology of grazing yaks between warm and cold seasons


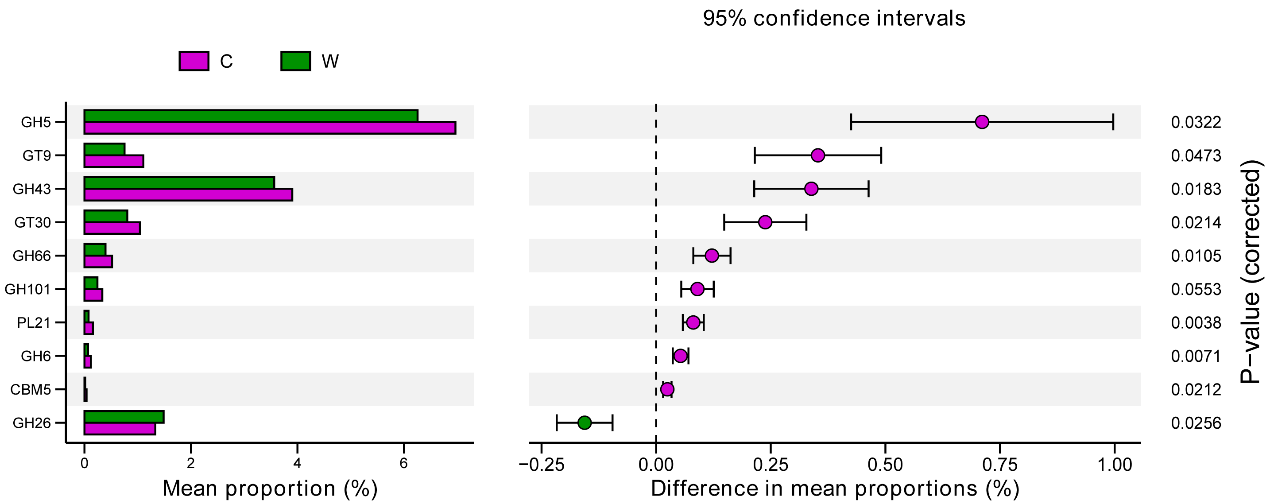


**Figure S2** Abundance of hemicellulose, cellulase, amylase, and acetylesterase genes of grazing yaks in warm and cold seasons


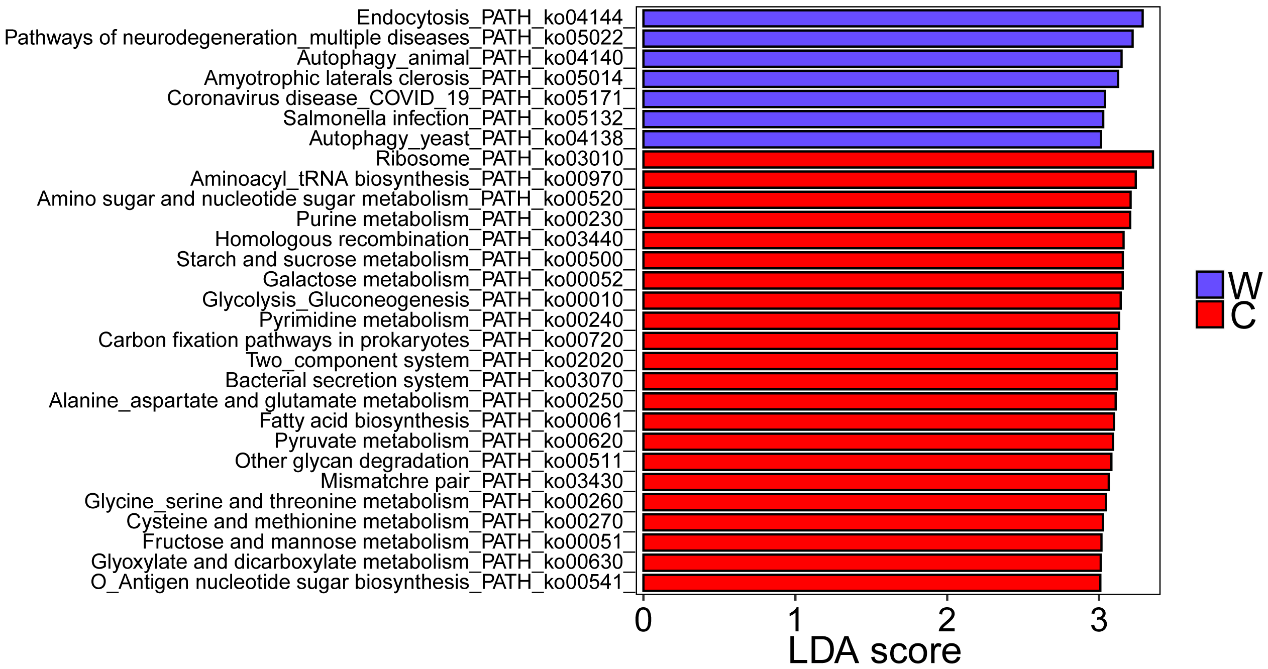


**Figure S3** Differential KEGG pathways of grazing yaks between warm and cold seasons at level 3. Differential KEGG pathways between yaks at warm and cold seasons as determined using LEfSe. Only the pathways that differed signiﬁcantly (*P* < 0.05) between yaks at warm and cold seasons with an LDA score > 3 are shown.


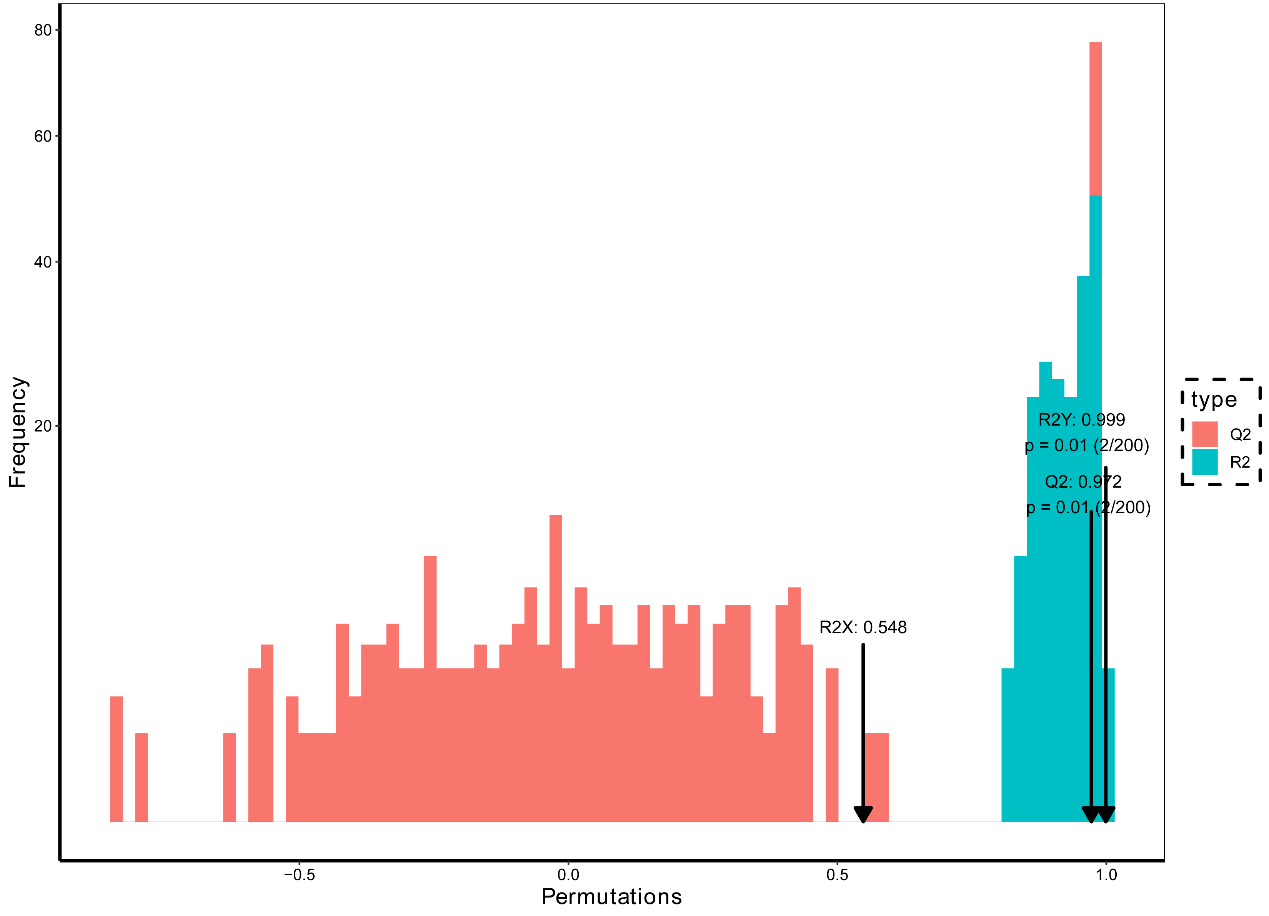


**Figure S4** Validation plot of the OPLS-DA model between warm and cold seasons


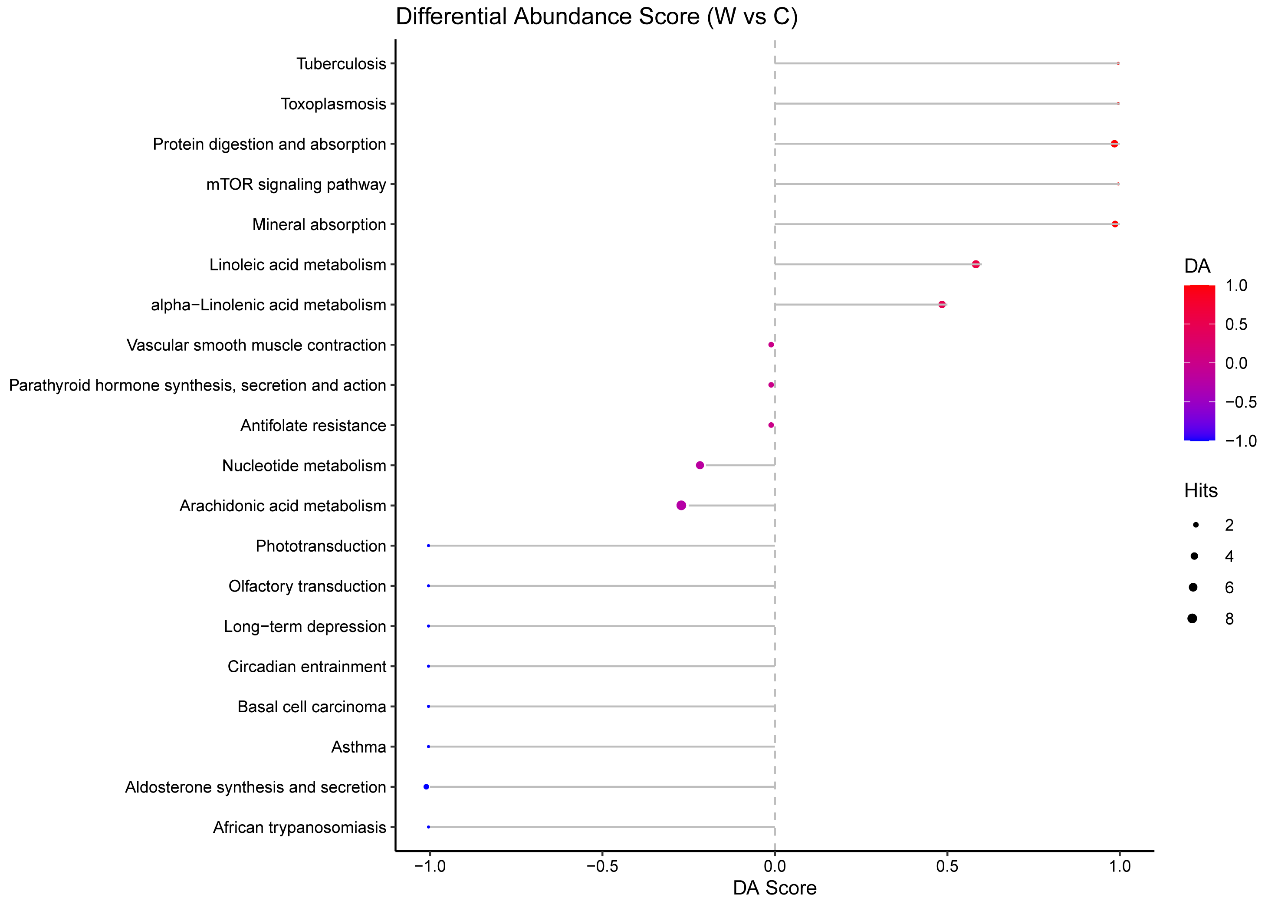


**Figure S5** Differential abundance score for metabolic pathways of grazing yaks between warm and cold seasons
